# Metacognitive beliefs about biased thinking condition the role of anxiety sensitivity in the somatic symptom–health anxiety pathway

**DOI:** 10.3389/fpsyt.2026.1766275

**Published:** 2026-04-16

**Authors:** Robin Bailey, Tiffany D. Carmichael, Alexander M. Penney

**Affiliations:** 1 Department of Psychiatry, University of Cambridge, Cambridge, United Kingdom; 2 Department of Psychology, MacEwan University, Edmonton, AB, Canada

**Keywords:** anxiety sensitivity, health anxiety, metacognitive beliefs, moderated moderation, somatic symptoms

## Abstract

**Background:**

Somatic symptoms are closely linked to health anxiety, and anxiety sensitivity is often described as a trait-like amplifier of responses to bodily sensations. Metacognitive beliefs operate at a higher-order level and may influence the conditions under which this amplification is observed.

**Methods:**

A cross-sectional survey was completed by 564 university students, who reported on somatic symptom burden (PHQ-15), anxiety sensitivity focused on physical concerns (ASI-3 Physical), metacognitive beliefs about biased thinking (MCQ-HA Biased Thinking), and health anxiety (SHAI). Pearson correlations and regression-based conditional process analyses were used to estimate a two-way moderation model and a three-way moderated moderation model (PROCESS Models 1 and 3) with 5,000 bootstrap samples and Johnson-Neyman probing.

**Results:**

Somatic symptoms, anxiety sensitivity, and biased-thinking beliefs were all positively associated with health anxiety. The two-way interaction between somatic symptoms and anxiety sensitivity was not clearly supported. A small but statistically significant three-way interaction indicated that anxiety sensitivity strengthened the somatic symptom to health anxiety association only at higher levels of biased-thinking beliefs, a range that applied to roughly one quarter of the sample.

**Conclusion:**

These findings provide preliminary support for the idea that anxiety sensitivity may act as a conditional vulnerability, increasing the impact of somatic symptoms on health anxiety primarily when metacognitive beliefs about biased thinking are high.

## Introduction

Hypochondriasis (1) or “health anxiety” is a psychological disorder that is characterised by specific worries and convictions about having or developing a serious illness, despite medical reassurance (2). Health anxiety is thought to be common, with rates ranging from 0.04-4.5% in the general population to higher percentages in medical settings such as 0.3–8.5% in GP surgeries and 12–20% in specialty hospital clinics (3). Recent research has indicated that health anxiety has been on the rise over the past 30 years (4).

Somatic symptoms, include a range of physical sensations, such as headaches, difficulty breathing, heart palpitations, fatigue, and gastrointestinal symptoms, and are highly prevalent across a range psychological disorder, e.g., depression and anxiety (5). Somatic symptoms are among the most common presenting problems in primary care (6).

Studies have shown that somatic symptoms have been strongly associated with health anxiety, with more somatic symptoms often accompanied by higher levels of HA (7–9). To explain the association between somatic symptoms and health anxiety several different psychological constructs have been proposed.

Anxiety sensitivity (AS) is a trait-like, dispositional individual difference reflecting the fear of anxiety-related bodily sensations (10). It functions as an ‘anxiety amplifier,’ such that individuals high in AS become alarmed by physiological arousal, which further intensifies anxiety (11–13). When such individuals experience anxiety, they are more likely to fear the symptoms and catastrophically misinterpret them, increasing the intensity of their emotional response. This construct encompasses fears of the somatic/physical, social, and cognitive consequences of anxiety (12, 14, 15). AS represents a stable cognitive predisposition to general fearfulness and serves as a vulnerability factor for anxiety psychopathology, playing a central role in both the onset and maintenance of various anxiety disorders (13, 16–20).

Anxiety sensitivity has been considered an important variable in the development and maintenance of health anxiety, (20–27) in particular the physical component (23, 28–31). In replicated studies (32, 33), the combination of high health anxiety and severe somatic symptoms was particularly strongly associated with anxiety sensitivity concerns, suggesting that anxiety sensitivity is especially relevant when somatic symptom burden is high. What seems crucial is that anxiety sensitivity moderates the intensity to which people experience and cope with physical sensations (34).

While cognitive models in particular offer theoretical explanations on the relationship between somatic symptoms, anxiety sensitivity, and health anxiety, they may overlook higher-order variables which may be critical in monitoring and controlling such interactions. One such higher-order variable that may significantly influence the relationship between somatic symptoms, anxiety sensitivity, and health anxiety is metacognition.

According to Self-Regulatory Executive Function (S-REF) model (35–37), metacognition is considered a top-down transdiagnostic construct across all psychopathologies (38). Metacognitive beliefs, particularly positive metacognitive beliefs, are theorised to increase the likelihood of engaging in the cognitive attentional syndrome (CAS). The CAS comprises a range of maladaptive thinking styles such as worry and rumination, threat monitoring and the use of maladaptive coping strategies (39). Excessive CAS activity can then lead to an increase and reinforcement of negative metacognitive beliefs such as worry is uncontrollable and dangerous (40).

Within health anxiety, metacognitive beliefs have been implicated as a significant predictor of health anxiety (41, 42). A recent meta-analysis of 23 studies (N = 9,229) found a medium association between positive metacognitions and health anxiety (r ≈.36) and a large association between negative metacognitions and health anxiety (r ≈.52), indicating that metacognitions are central pathological factors in health anxiety rather than incidental correlates (43). Complementing this, a broader systematic review showed that metacognitive beliefs are a common transdiagnostic factor across somatic distress and health anxiety (44). Metacognition has also been shown to moderate the effects of cognition on health anxiety, both cross-sectionally and prospectively (42, 45). Within the metacognitive model, anxiety sensitivity can be understood as operating primarily at the cognitive level, reflecting appraisal and schema-based interpretations of physical sensations. By contrast, metacognitive beliefs sit at a higher-order level concerned with monitoring and regulating cognitive responses to perceived threat, including the interpretation of bodily cues. Consistent with this integration, Gorday and Bardeen (46) reported that metacognitive beliefs moderated the association between anxiety sensitivity and anxiety, such that the association between anxiety sensitivity and anxiety was stronger at higher levels of both negative and positive metacognitive beliefs. In health anxiety, three particular metacognitive beliefs have emerged as being particularly problematic, these are ‘Beliefs that Thoughts cause Illness’ (MCQ-HAC), e.g., ‘Thinking negatively can increase my chances of disease’; ‘Beliefs about Biased Thinking’ (MCQ-HAB) e.g., ‘Worrying about my health will help me cope’ or ‘Thinking positively about my health will tempt fate and I will become ill.’; and ‘Beliefs that Thoughts are Uncontrollable’ (MCQ-HAU), e.g., ‘I have no control over thinking about my health’. As mentioned, recent meta-analytic work indicates that positive metacognitive beliefs are reliably associated with health anxiety (43), and within this broader class of beliefs, health-specific beliefs about biased thinking have been shown prospectively to play a causal role in the development of health anxiety (47). Given that beliefs about biased thinking are a type of positive metacognitive belief, which are thought to initiate the worry process, it can be hypothesised that these beliefs are an important health-related metacognitive factor that shapes how a pre-existing sensitivity to bodily sensations (anxiety sensitivity) is expressed in response to somatic symptoms. These bias beliefs consist of two interconnected elements, positive beliefs about negative thinking and negative beliefs about positive thinking. First, individuals may hold beliefs about the usefulness of thinking *negatively* about symptoms as a coping strategy, such as “Thinking the worst about symptoms will keep me safe.” These beliefs can lead to sustained negative thinking (worry/rumination), which maintains a sense of threat and hypervigilance toward health-related information, including bodily sensations.

Second, individuals may hold beliefs regarding the potential danger of thinking *positively* about symptoms, such as “Thinking positively about my health will tempt fate, and I will become ill.” These beliefs can lead individuals to actively avoid or dismiss positive thoughts about their health and normal bodily sensations. Despite being in a generally positive state of mind or experiencing positive or in fact no physical symptoms, they may continue to believe that worrying about somatic symptoms and health is necessary to prevent illness. A number of experimental studies have found that individuals with high levels of anxiety sensitivity (34) and high level of metacognitive beliefs (48) find positive health-related information to be threatening.

In the moderation-based studies (42, 46, 47) the authors have looked only at two-way interactions i.e. metacognition * catastrophic misinterpretation or metacognition * anxiety sensitivity. However, the additional role of somatic symptoms in these interactions has not been explored. It therefore remains unclear whether the moderation of the association between somatic symptoms and health anxiety by anxiety sensitivity alone is sufficient to account for individual differences in health anxiety or whether this relationship is dependent upon an additional higher-order variable, such as metacognition. This raises the possibility of a three-way interaction, wherein metacognitive beliefs may further condition the interplay between somatic symptoms, anxiety sensitivity, and health anxiety. Consistent with the conclusions of Ivan et al. (43), this moves beyond simple associations to examine whether metacognitions condition the impact of both symptoms and cognitive vulnerability factors on health anxiety.

In this study, we propose that beliefs about bias thinking may act as a second-order moderator, namely, the moderating effect of anxiety sensitivity on somatic symptoms and health anxiety is subject to these metacognitive beliefs. As such we hypothesised.

1. Somatic symptoms, anxiety sensitivity, and metacognitive beliefs will be positively associated with health anxiety.
2. The relationship between somatic symptoms and health anxiety would be moderated by anxiety sensitivity.
3. The moderated relationship between somatic symptoms and health anxiety by anxiety sensitivity will be stronger in the presence of metacognitive beliefs about bias thinking, creating a positive significant three-way interaction.

## Method

### Participants

The data for the present study were drawn from the undergraduate sample reported by Carmichael and Penney (49). In that paper, the authors examined mediation pathways from negative and positive affect to health anxiety. Here, we address different hypotheses concerning moderated moderation between somatic symptoms (PHQ-15), anxiety sensitivity physical concerns (ASI-P), and metacognitive beliefs about biased thinking (MCQ-B).

The sample consisted of 564 individuals at a Canadian university. Participants were a young, predominantly female non-clinical sample (72.3%) and had a mean age of 21.83 years old (*SD* = 5.04). No inclusion or exclusion criteria were used.

The MacEwan University Research Ethics Board (File Number 101769) approved this study. Students voluntarily self-selected to take part via an online participant management portal and received 2% course credit for participating.

Participants were also asked whether they had previously received a mental health diagnosis from a medical doctor, counsellor, or other professional. These data were self-reported and were therefore treated as descriptive only. Reported diagnoses were not mutually exclusive and included anxiety (11.9%), generalised anxiety disorder (4.1%), PTSD (1.8%), depression (9.4%), and ADHD (5.1%). These figures should be interpreted cautiously because diagnoses were not independently verified.

The study was completed online through Qualtrics. Participants were presented with a consent form, and informed consent was obtained. They first completed a demographic characteristics questionnaire, then completed the remaining measures in a randomized order, followed by an online debriefing form.

### Measures

#### Short health anxiety inventory

The Short Health Anxiety Inventory (SHAI; 50) is an 18-item self-report questionnaire designed to assess health anxiety (HA). Each item presents four statements, with responses scored from 0 to 3, resulting in a total score ranging from 0 to 54. The SHAI assesses cognitive, emotional, and behavioural features of health anxiety, including illness preoccupation, anticipated negative consequences of illness, and reassurance-seeking, rather than health-related cognitions alone. The SHAI is effective in distinguishing between clinical and subclinical levels of health anxiety, regardless of the respondent’s actual physical health status (51). Studies have consistently demonstrated the SHAI’s strong construct validity, making it a reliable and psychometrically robust tool for evaluating health anxiety (52). Additionally, its validity and reliability have been confirmed across various clinical settings, further supporting its use in both research and practice (53).

#### Patient health questionnaire-15

The Patient Health Questionnaire-15 (PHQ-15) is a commonly used measure for identifying people with a high level of somatic symptom burden. It focuses on frequent physical complaints, including fatigue, gastrointestinal problems, musculoskeletal pain, and cardiopulmonary symptoms, assessed over the previous four weeks across 15 items. Responses are scored from 0 (not bothered at all) to 2 (bothered a lot), producing a total score between 0 and 30. Higher totals indicate greater symptom burden and are typically interpreted using the following categories: no/minimal (0–4), low (5–9), medium (10–14), and high (15–30) (6). The PHQ-15 shows strong internal consistency, with a reported Cronbach’s α of approximately 0.80. Given its solid psychometric performance, it is often recommended for use in large-scale research (54). Validation work across varied populations further supports its value for both clinical assessment and research applications (55).

#### Anxiety sensitivity index-3

The Physical Concerns subscale of the Anxiety Sensitivity Index-3 (ASI-P; 12) was used to assess anxiety sensitivity. The ASI-P consists of 6 items rated from 0 (“very little”) to 4 (“very much”), yielding total scores from 0 to 24. It indexes a relatively stable, trait-like fear of the physical consequences of bodily sensations (e.g., concerns that palpitations indicate serious illness or that breathlessness signals a medical crisis), rather than the frequency or intensity of those sensations themselves. Higher scores reflect greater dispositional fear of benign somatic sensations. The ASI-P has demonstrated good internal consistency and convergent validity and has been associated in longitudinal research with heightened risk for anxiety symptoms and disorders, supporting its role as a vulnerability factor rather than a simple state marker of current distress.

#### Metacognition questionnaire-health anxiety

The Metacognition Questionnaire-Health Anxiety (MCQ-HA; 56) is a 14-item measure designed to assess metacognitive beliefs related to health-related thoughts. The MCQ-HA comprises three subscales: (1) ‘Beliefs about Biased Thinking’ (MCQ-B; 5 items), which captures positive beliefs about negative thinking and negative beliefs about positive thinking; (2) ‘Beliefs that Thoughts Can Cause Illness’ (MCQ-C; 5 items); and (3) ‘Beliefs about Uncontrollability of Thoughts about Illness’ (MCQ-U; 4 items). Each item is rated on a scale from 0 to 4. The subscale scores range from 0 to 20 for MCQ-B and MCQ-C, and from 0 to 16 for MCQ-U. The MCQ-HA has demonstrated good internal consistency and convergent validity, making it a reliable tool for assessing metacognitive beliefs in the context of health anxiety (56, 57). Its validity has been supported by research linking metacognitive beliefs to the maintenance of health anxiety, suggesting its relevance in both clinical assessment and therapeutic intervention (56). Only the MCQ-B was used in this study.

### Data analysis

The analytic strategy employed was a moderated moderation model. Anxiety sensitivity (AS) is a trait-like predisposition rather than a state arising from symptoms, making it theoretically and statistically appropriate to model as a moderator of the relationship between somatic symptoms and health anxiety, rather than as a mediator. Higher-order metacognitive beliefs were included as a second-order moderator to examine whether they condition the effect of AS on the somatic symptom to health anxiety pathway, consistent with the hierarchical structure proposed by metacognitive theory (36, 37). The moderated moderation approach (58) offers a statistically precise way to model this nested regulatory structure, allowing us to test whether anxiety sensitivity is associated with a stronger somatic symptom to health anxiety association under higher levels of metacognitive beliefs. In this context the analytic method is theoretically motivated, given the hierarchical structure proposed by metacognitive theory. Given the cross-sectional design, these models estimate conditional associations rather than causal pathways, and the terms ‘moderator’ and ‘amplifying effect’ are used in this statistical sense.

Pearson’s correlation analyses were conducted using SPSS version 22.0. To test Hypothesis 2, we first examined a two-way moderation model (PROCESS Model 1; 58) to assess whether anxiety sensitivity (W) moderated the relationship between somatic symptoms (X) and health anxiety (Y). To test our primary hypothesis (Hypothesis 3), we then employed a three-way moderated moderation model (PROCESS Model 3; 58), with metacognitive beliefs about biased thinking (Z) specified as a second-order moderator. All models were estimated with 5,000 bootstrap samples. Variables were analysed on their original scales without mean-centring, and interaction terms were computed from the raw variables. Johnson–Neyman probing was used to identify regions of significance.

The three-way model can be expressed as:


$$\text{Y}=B_{0}+B_{1}X+B_{2}Z+B_{3}W+B_{4}\left(X\times W\right)+B_{5}\left(X\times Z\right)+B_{6}\left(Z\times W\right)+B_{7}\left(X\times Z\times W\right)+\text{ϵ}$$

where Y = health anxiety, X = somatic symptoms, W = anxiety sensitivity, and Z = metacognitive beliefs about biased thinking.

This analytic strategy allowed us to evaluate direct effects, two-way interactions (H2), and the hypothesised three-way interaction (H3).

## Results

### Scale descriptives and intercorrelations

**Table 1** presents the mean, standard deviation, range, and Cronbach’s alpha for each scale.

**Table 1:** Scale descriptive statistics.

Measure	Mean	SD	Observed range	Cronbach’s alpha
SHAI	18.02	7.80	2–48	0.87
ASI-P	6.39	5.53	0–24	0.86
PHQ-15	10.30	5.30	0-26	0.81
MCQ-B	6.98	2.47	5–17	0.78

SHAI ,  Short Health Anxiety Inventory; ASI-P ,  Anxiety Sensitivity Index-3 – Physical concerns subscale; PHQ-15 ,  Patient Health Questionnaire-15; MCQ-B ,  Metacognition Questionnaire-Health Anxiety - Beliefs about biased thinking subscale.

Correlation results (**Table 2**) showed positive and significant correlations between all variables and health anxiety. This is in line with previous research which identifies these variables being associated with health anxiety.

**Table 2:** Bivariate correlations among study variables.

Variable	1	2	3	4
1. SHAI	1.00			
2. PHQ-15	.516 (*p* <.001)	1.00		
3. ASI-P	.518 (*p* <.001)	.441 (*p* <.001)	1.00	
4. MCQ-B	.325 (*p* <.001)	.128 (*p* = .002)	.388 (*p* <.001)	1.00

Entries are Pearson’s r with two-tailed *p* shown in parentheses. SHAI, Short Health Anxiety Inventory; PHQ-15, Patient Health Questionnaire Somatic Symptom scale; ASI-P, Anxiety Sensitivity Index-3 – Physical concerns subscale; MCQ-B, Metacognition Questionnaire-Health Anxiety - Beliefs about biased thinking subscale.

### Moderated moderation effects analyses

To assess hypothesis 2, a two-way moderation analysis (PROCESS Model 1) was first conducted to examine whether anxiety sensitivity (W) moderated the association between somatic symptoms (X) and health anxiety (Y). The overall model was statistically significant, *F*(3, 557) = 111.69, *p* <.001, accounting for 37.6% of the variance in health anxiety (*R²* = .3756). Significant main effects were found for both somatic symptoms (*B* = 0.4064, p <.001) and anxiety sensitivity (*B* = 0.3310, p = .0019), each positively associated with health anxiety. However, the interaction between somatic symptoms and anxiety sensitivity (XW) was marginally non-significant (*B* = 0.0157, *p* = .0511), suggesting that the central prediction, that the relationship between somatic symptoms and health anxiety is conditional upon anxiety sensitivity, was not robustly supported in this dataset.

**Figure 1:**
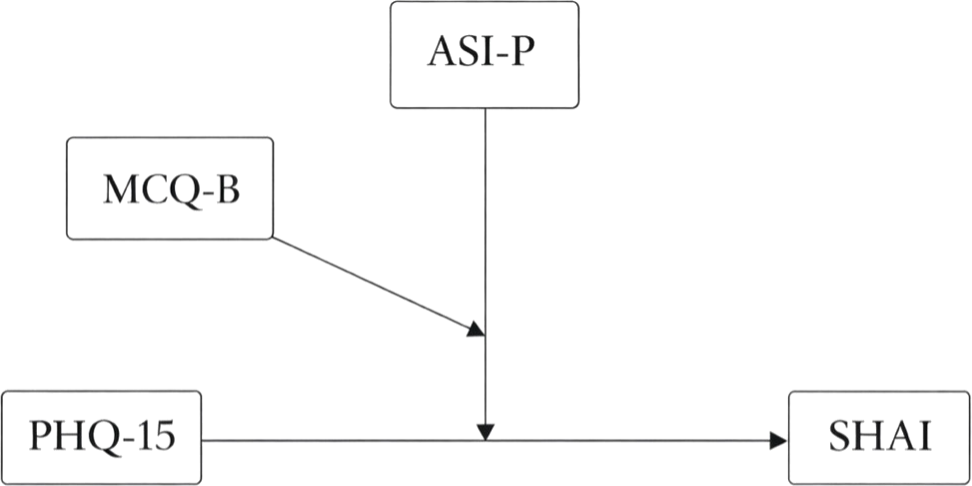
Examined moderated moderation.

To test our main hypothesis, a three-way moderated moderation model (**Figure 1**) was then conducted using (58) PROCESS macro (Model 3) to examine whether anxiety sensitivity (W) and beliefs about biased thinking (Z) moderated the relationship between somatic symptoms (X) and health anxiety (Y). The model was statistically significant, *F*(7, 551) = 56.12, p <.001, and accounted for 41.6% of the variance in health anxiety (*R²* = .4162).

**Table 3** presents the regression coefficients. Significant main effects were observed for somatic symptoms (*B* = 0.6772, p = .0119), anxiety sensitivity (*B* = 1.2110, p <.001), and beliefs about biased thinking (*B* = 1.4123, p = .0015), each independently associated with higher levels of health anxiety.

**Table 3:** Regression coefficients for the three-way moderated moderation model predicting health anxiety.

Predictor	B	SE	t	p	LLCI	ULCI
Constant	1.6766	2.847	0.5889	0.5562	-3.9157	7.2689
PHQ-15 (Somatic Symptoms)	0.6772	0.2684	2.5228	0.0119	0.1499	1.2044
ASI-P (Anxiety Sensitivity)	1.211	0.295	4.1054	0.0	0.6316	1.7905
PHQ-15 × ASI-P	-0.0387	0.0234	-1.6524	0.099	-0.0847	0.0073
MCQ-B (Beliefs about Biased Thinking)	1.4123	0.4415	3.1991	0.0015	0.5451	2.2794
PHQ-15 × MCQ-B	-0.0425	0.0401	-1.0596	0.2898	-0.1211	0.0362
ASI-P × MCQ-B	-0.129	0.0368	-3.504	0.0005	-0.2013	-0.0567
PHQ-15 × ASI-P × MCQ-B	0.0071	0.003	2.4191	0.0159	0.0013	0.0129

Among the interaction terms, the two-way interaction between somatic symptoms and anxiety sensitivity (XW) was not significant (*B* = −0.0387, 95% *CI* [−0.0847, 0.0073], p = .099), indicating that the association between somatic symptoms and health anxiety did not vary significantly across levels of anxiety sensitivity. Similarly, the interaction between somatic symptoms and beliefs about biased thinking (XZ) was not significant (*B* = −0.0425, 95% *CI* [−0.1211, 0.0362], p = .290), suggesting no conditional effect of metacognitive beliefs on this pathway.

By contrast, the interaction between anxiety sensitivity and metacognitive beliefs (WZ) was significant and negative (*B* = −0.1290, 95% *CI* [−0.2013, −0.0567], *p* <.001), indicating that as metacognitive beliefs increased, the direct relationship between anxiety sensitivity and health anxiety became weaker. Statistically, this reflects a significant attenuation of the W to Y effect at higher values of Z.

The three-way interaction (XWZ) between somatic symptoms, anxiety sensitivity, and metacognitive beliefs was statistically significant (*B* = 0.0071, 95% *CI* [0.0013, 0.0129], *p* = .0159). This indicates that the extent to which anxiety sensitivity moderates the relationship between somatic symptoms and health anxiety depends on the level of metacognitive beliefs. In other words, metacognitive beliefs may alter the moderating role of anxiety sensitivity, shaping how somatic symptoms translate into health anxiety. The inclusion of this interaction explained an additional 0.62% of the variance in health anxiety (Δ*R*² = .0062). In moderated regression models, Δ*R*² reflects the incremental effect size associated with the interaction term. This magnitude would typically be interpreted as a small effect.

Taken together, the negative WZ term and the positive three-way interaction indicate a shift in the role of anxiety sensitivity as metacognitive beliefs increase. At higher levels of metacognitive beliefs, anxiety sensitivity appears less important as a simple stand-alone predictor of health anxiety and more important as part of a conditional configuration with somatic symptoms. In this configuration, anxiety sensitivity appears to amplify the impact of somatic symptoms on health anxiety specifically when metacognitive beliefs about biased thinking are high.

### Probing the three-way interaction

To further explore the significant three-way interaction between somatic symptoms (X), anxiety sensitivity (W), and beliefs about biased thinking (Z), we conducted follow-up analyses examining the conditional X × W interaction at specific values of Z. These values corresponded to the 16th percentile (Z = 5), 50^th^ percentile (Z = 6), and 84th percentile (Z = 9) of the MCQ-B distribution.

**Figure 2:**
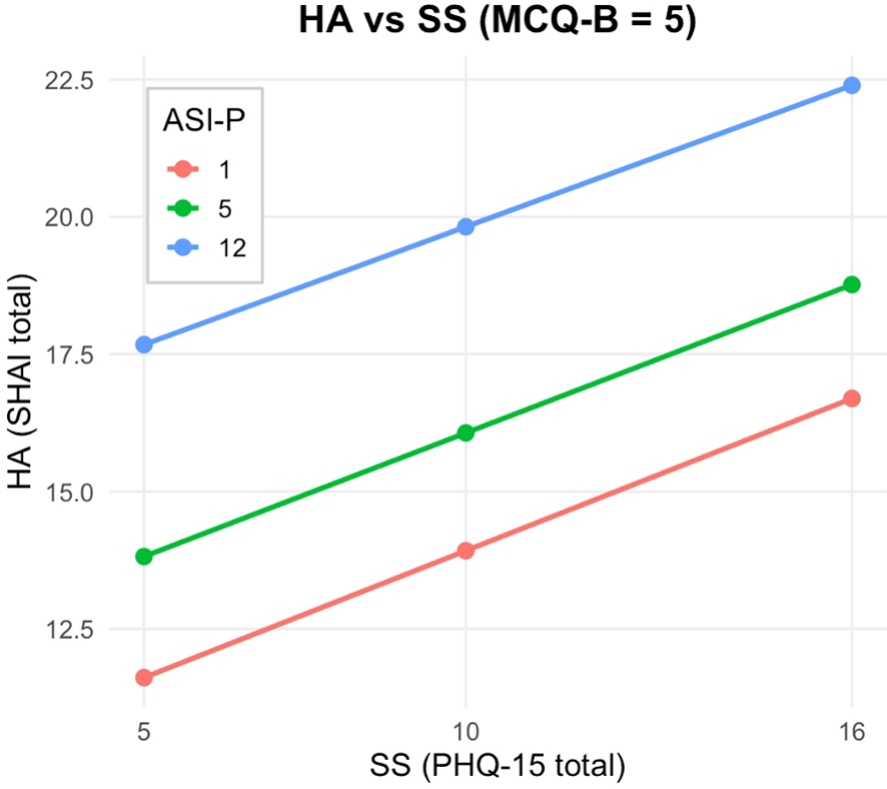
Johnson-Neyman plot and conditional effect estimates for the PHQ-15 × ASI-P interaction at low levels of metacognitive beliefs about biased thinking (MCQ-B; Z = 5). At this level, the interaction between somatic symptoms and anxiety sensitivity is non-significant (Effect = −0.0030, p = .7906), indicating that anxiety sensitivity does not moderate the association between somatic symptoms and health anxiety.

**Figure 3:**
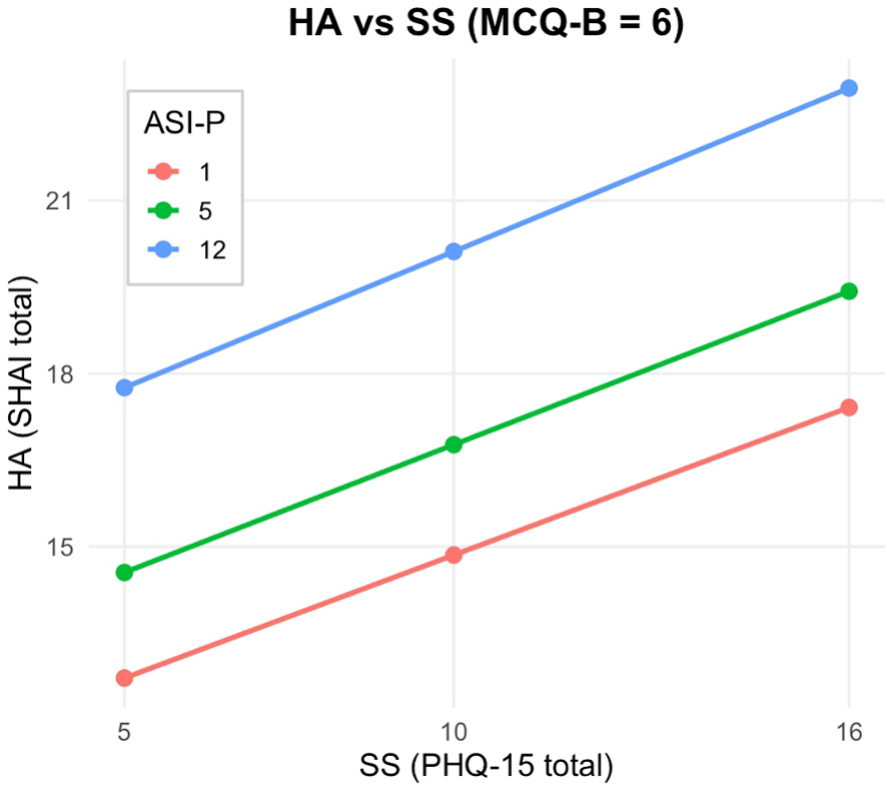
Johnson-Neyman plot and conditional effect estimates for the PHQ-15 × ASI-P interaction at median levels of metacognitive beliefs about biased thinking (MCQ-B; Z = 6). The interaction remains non-significant (Effect = 0.0041, p = .6708), suggesting that anxiety sensitivity still does not appreciably alter the somatic symptom–health anxiety association at typical levels of MCQ-B.

**Figure 4:**
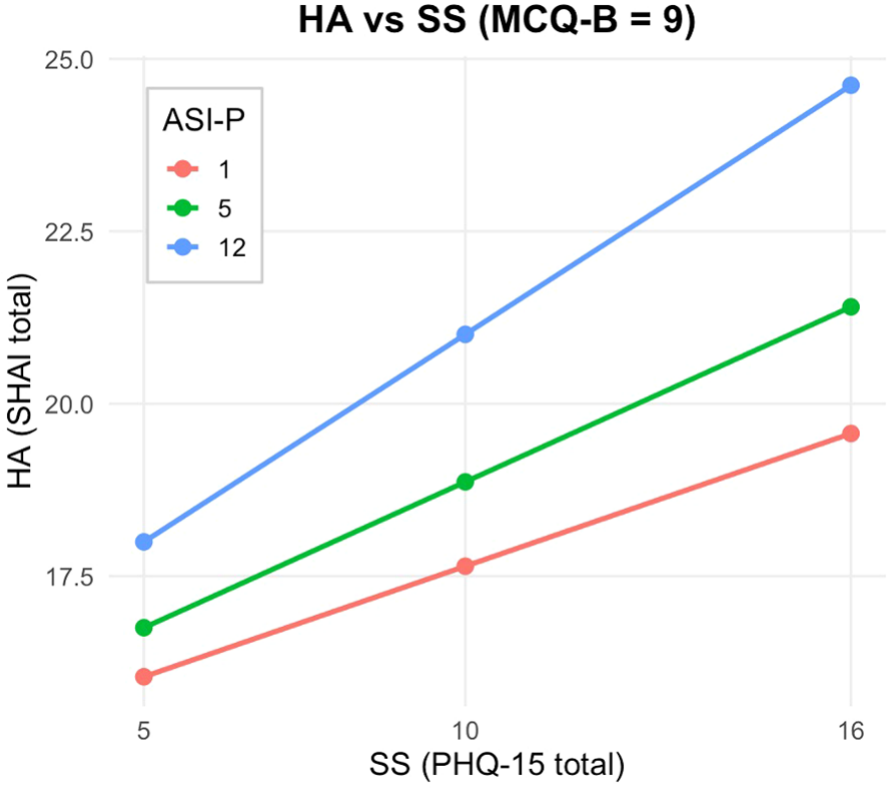
Johnson-Neyman plot and conditional effect estimates for the PHQ-15 × ASI-P interaction at high levels of metacognitive beliefs about biased thinking (MCQ-B; Z = 9). At this level, the interaction between somatic symptoms and anxiety sensitivity is statistically significant and positive (Effect = 0.0256, p = .0123), indicating that anxiety sensitivity amplifies the effect of somatic symptoms on health anxiety.

At low levels of beliefs about biased thinking (MCQ-B; Z = 5; **Figure 2**), the interaction between somatic symptoms and anxiety sensitivity was non-significant (*Effect* = −0.0030, *p* = .7906), indicating that anxiety sensitivity did not moderate the relationship between somatic symptoms and health anxiety. At the median level of MCQ-B (Z = 6; **Figure 3**), the interaction remained non-significant (*Effect* = 0.0041, *p* = .6708). By contrast, at high levels of metacognitive beliefs (Z = 9; **Figure 4**), the interaction became statistically significant and positive (*Effect* = 0.0256, *p* = .0123), suggesting that when individuals strongly endorse beliefs that their thinking is useful or protective, anxiety sensitivity amplifies the effect of somatic symptoms on health anxiety.

These findings were further clarified by a Johnson–Neyman analysis (**Figures 2**–**4**), which identified the region of significance for the X × W interaction across the full range of Z. Results indicated that the conditional interaction between somatic symptoms and anxiety sensitivity became statistically significant when beliefs about biased thinking exceeded a value of Z = 7.92. Approximately 27% of participants scored above this threshold, indicating that the interactive impact of somatic symptoms and anxiety sensitivity on health anxiety emerges primarily in individuals with high levels of metacognitive beliefs.

Taken together, these results are consistent with the view that anxiety sensitivity functions as a moderator of the somatic symptom-health anxiety relationship primarily in individuals who strongly believe their thinking is beneficial or protective, which fits with the metacognitive model.

## Discussion

We examined whether the moderating effect of anxiety sensitivity on the somatic symptoms to health anxiety pathway depends on metacognitive beliefs about biased thinking. The findings suggest that beliefs about biased thinking may help define the conditions under which anxiety sensitivity is most relevant. In practical terms, this pattern suggests that metacognitive beliefs about biased thinking appear to be associated with when anxiety sensitivity conditions the strength of the association between somatic symptoms and health anxiety.

The zero-order correlations showed that somatic symptoms, anxiety sensitivity, and metacognitive beliefs were each positively associated with health anxiety, which corresponds with prior work highlighting these variables in the maintenance of health anxiety (9, 44, 59).

In relation to our second hypothesis, the two-way interaction between somatic symptoms and anxiety sensitivity was not significant. This suggests that anxiety sensitivity may not, on its own, reliably alter how symptoms relate to health anxiety. The finding contrasts with reports of a two-way effect in some previous studies (e.g., 29, 33, 60) and with aspects of cognitive accounts that treat anxiety sensitivity as a general vulnerability factor (21, 53). A potential explanation for this is that part of what appears as an anxiety sensitivity effect in simpler health anxiety models may reflect a mix of adaptive vigilance and psychopathology. Interoceptive changes can indicate normal physiological variation or, at times, legitimate medical risk, and sensitivity may support proportionate help-seeking and self-care (61). For example, unexplained chest pain might indicate a cardiac issue, and persistent nausea may warrant investigation for gastrointestinal or hepatic conditions. Heightened physical sensitivity and fear of symptoms may therefore sometimes prompt appropriate protective behaviours, such as seeking medical advice, which are consistent with survival and public health guidance. Against this backdrop, our findings suggest that additional variables (i.e. metacognitive beliefs) may be responsible for when anxiety sensitivity to physical symptoms leads to further sustained worry and threat monitoring and ultimately pathological health anxiety.

Our primary hypothesis received support. A significant three-way interaction indicated that the anxiety sensitivity by symptoms effect emerges only when beliefs about biased thinking are elevated. Probing showed that at low or median levels of these beliefs, anxiety sensitivity did not strengthen the symptom to health anxiety pathway, whereas at high levels it did. Johnson-Neyman probing showed that the conditional anxiety sensitivity by symptoms effect became statistically significant only above the observed threshold on beliefs about biased thinking in this sample (Z ≈ 7.92), with approximately 27 percent of participants scoring above that point. In short, metacognitive bias-beliefs determine when anxiety sensitivity meaningfully conditions the symptom-health anxiety relationship, rather than exerting a uniform influence across individuals.

The observed two-way negative anxiety sensitivity by metacognition interaction helps clarify this pattern. As beliefs about biased thinking increase, the simple stand-alone association between anxiety sensitivity and health anxiety weakens on average. Conceptually, these findings may indicate, when the metacognitive rule set is strong, health anxiety is less about a general tendency to fear sensations and more about whether those metacognitive rules are engaged by symptoms. Once symptoms are present and beliefs authorise worry and monitoring as useful or protective, anxiety sensitivity becomes relevant again, now functioning more like an amplifier of the symptom pathway rather than a global predictor.

These findings are consistent with metacognitive theory, in which positive metacognitive beliefs initiate and maintain the cognitive attentional syndrome, including worry, threat monitoring, and maladaptive coping (37). More broadly, this kind of hierarchical pattern has parallels in recent computational accounts of anxiety, which emphasise metacognitive beliefs in shaping when anxious responses escalate into pathology (e.g., 62).

The wider literature adds to the importance of biased-thinking beliefs. Prospectively, Bailey and Wells (47) showed that these beliefs uniquely predicted health anxiety six months later, over and above cognitive and personality variables, and prospectively moderated the relationship between catastrophic misinterpretation and health anxiety.

In Ivan et al.’s (43) meta-analysis, positive metacognitive beliefs, including biased-thinking beliefs, showed medium to large associations with health anxiety. These effect sizes are numerically larger than those reported for GAD and OCD in an earlier transdiagnostic meta-analysis of dysfunctional metacognition (38), and larger than effects observed in physical-illness samples with anxiety or depression (63), which reinforces the particular relevance of these beliefs in health anxiety.

Interventional evidence is consistent with this interpretation. In a randomised controlled trial of metacognitive therapy for health anxiety, targeting biased-thinking beliefs as part of an MCT protocol produced substantial clinical gains, with approximately 80 percent of participants meeting recovery criteria at post-treatment and follow-up (64).

Overall, metacognition appears to be associated with when lower-level cognitive vulnerabilities are most likely to amplify the impact of somatic symptoms on health anxiety. Anxiety sensitivity may be best understood as a conditional vulnerability that modulates the translation of symptoms into health anxiety, primarily when biased-thinking beliefs are high enough to engage and sustain the cognitive attentional syndrome.

Targeting biased-thinking beliefs should both reduce the baseline influence of anxiety sensitivity and prevent anxiety sensitivity from switching into amplifier mode when symptoms arise. This provides a coherent rationale for prioritising these metacognitive beliefs early in treatment and is consistent with observed treatment gains in metacognitive therapy for health anxiety.

## Limitations and conclusions

The cross-sectional and non-clinical design limits causal inference and clinical generalisability. Participants were a non-clinical sample young and predominantly female. All measures were self-report, so shared method variance and overlapping item content may inflate associations between somatic symptoms, anxiety sensitivity, metacognition, and health anxiety. In addition, the three-way interaction accounted for a modest increase in explained variance, so the effect, while theoretically informative, is statistically small. Future work should test temporal ordering prospectively, evaluate clinical samples, and examine whether experimentally altering biased-thinking beliefs causally disrupts the symptom by anxiety sensitivity pathway.

In conclusion, the present study shows that we did not observe evidence that anxiety sensitivity uniformly amplifies the symptom to health anxiety association across the sample. Rather, its moderating influence is conditional on metacognitive beliefs about biased thinking. When these beliefs are high, anxiety sensitivity strengthens the link between somatic experiences and health anxiety. This three-way pattern supports the central proposition of metacognitive theory in health anxiety and identifies beliefs about biased thinking as a pivotal target for intervention.

## Data Availability

The aggregated data supporting the conclusions of this article will be made available by the authors, without undue reservation.
